# Synthesis, Screening and Pharmacokinetic Evaluation of Potential Prodrugs of Bupropion. Part One: *In Vitro* Development

**DOI:** 10.3390/ph7050595

**Published:** 2014-05-14

**Authors:** Paul Matthew O’Byrne, Robert Williams, John J. Walsh, John F. Gilmer

**Affiliations:** School of Pharmacy and Pharmaceutical Sciences, University of Dublin, Trinity College, Dublin 1, Ireland; E-Mails: robert.williams@lifescientific.com (R.W.); jjwalsh@tcd.ie (J.J.W.); gilmerjf@tcd.ie (J.F.G.)

**Keywords:** bupropion, bioprecursor, prodrug, microsomes, plasma, *in vitro*, metabolism

## Abstract

In general, prodrugs are developed to circumvent deficiencies associated with the absorption, distribution, metabolism, excretion or toxicological (ADMET) profile associated with the active drug. In our study, we select bupropion, a drug with broad pharmacology incorporating dopaminergic, noradrenergic, nicotinic and cytokine modulation properties, but which is rapidly metabolized *in vivo*. We exploited its carbonyl and secondary amine functionality to facilitate the synthesis of bioprecursor prodrug forms with the sole objective of identifying analogues with enhanced properties over bupropion. A range of analogues were synthesized, ranging from *N-*methyl, *N-*benzyl, oximes, enol acetate and ether forms to examples where both functional groups were utilized to form oxadiazine, oxadiazinone, oxazolone and acetylated derivatives. We then developed an *in vitro* metabolic screen to simulate the human oral delivery route for these analogues. The selection of media in the screens contained a variety of pH, enzymatic and co-factor systems which mimic metabolic *in vivo* environments that drugs encounter when delivered orally. By coupling our *in vitro* screening tool to a selective hyphenated technique such as LC-MS, we were able to quickly select potential prodrugs for further *in vitro* and *in vivo* development. From the data generated, the *N-*alkylated bupropion analogues were shown to have the highest potential to act as bioprecursor prodrugs of bupropion.

## 1. Introduction

Usually in prodrug design and discovery, a single functional group is modified with a metabolic vector in mind. The modification will ideally impart some new chemical characteristic to the compound that mitigates some pharmaceutical or toxicological issue. Bioactivation potential is then assessed using an *in vitro* model of the metabolic vector. In the study described here we adopted a different approach with bupropion where we systematically modified each of its functional groups. As we did not start with a single target metabolic vector, we developed a matrix approach that could mimic the conditions encountered following oral administration. The matrix we developed included simulated human gastric and intestinal fluids, human intestinal and liver microsomes and plasma. We feel this systematic approach has merit for identifying congeners and prodrugs and for predicting drug metabolism.

Bupropion was selected as a model compound as there is little data reported on prodrug forms of this compound in the literature. Bupropion is an interesting candidate to study owing to its broad pharmacology, its numerous therapeutic targets and its chemistry. It is a substituted cathinone, an *α*-aminoketone that acts primarily at four therapeutic sites. It is a dopamine and norepinephrine reuptake inhibitor [1] and a nicotinic acetylcholine receptor antagonist [2]. It has also been shown to modulate specific cytokine levels implicated in inflammatory diseases [3].

**Figure 1:**
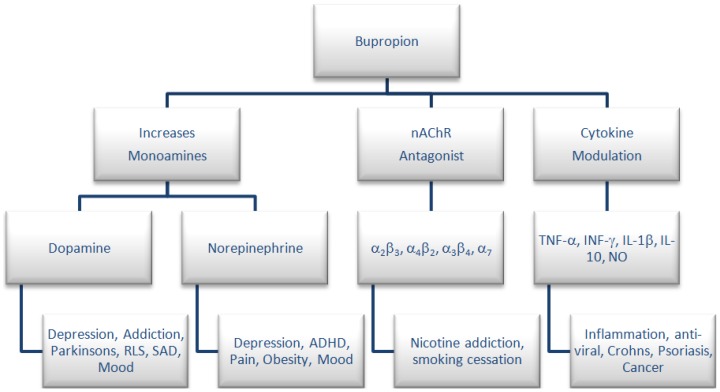
Pharmacology and therapeutic uses for bupropion.

Numerous studies have been carried out to examine the effectiveness of bupropion in a tobacco-use cessation therapy [4,5], in Crohn’s disease [3,6,7,8,9,10,11,12,13,14,15], pain [16,17,18], attention deficit hyperactivity disorder [19], restless leg syndrome [20] and seasonal affective disorder [21,22] as well as an adjuvant treatment for multiple myeloma [9,12]. A graphical representation of the pharmacological activity and therapeutic uses of bupropion is presented in Figure 1.

The absence of literature data on a prodrug approach for bupropion is unusual for such a widely used drug, but some attempts have been made to develop prodrug approaches for its active metabolite hydroxybupropion. A transdermal prodrug delivery approach has been developed for percutaneous absorption of hydroxybupropion [23]. A codrug approach for delivery of hydroxybupropion has also been developed [24]. A codrug is one that when metabolized forms two active compounds *in vivo*. This approach is similar to a prodrug approach as both require the body to metabolize the parent entities into their active forms *in vivo*. Diethylpropion is an *N-*ethyl prodrug of ethylpropion. Ethylpropion is an α-aminoketone related to bupropion, used as an appetite suppressant to treat obesity. Diethylpropion is *N-*de-ethylated *in vivo* to ethcathinone which is a selective norepinephrine releasing agent [25].

**Scheme 1:**
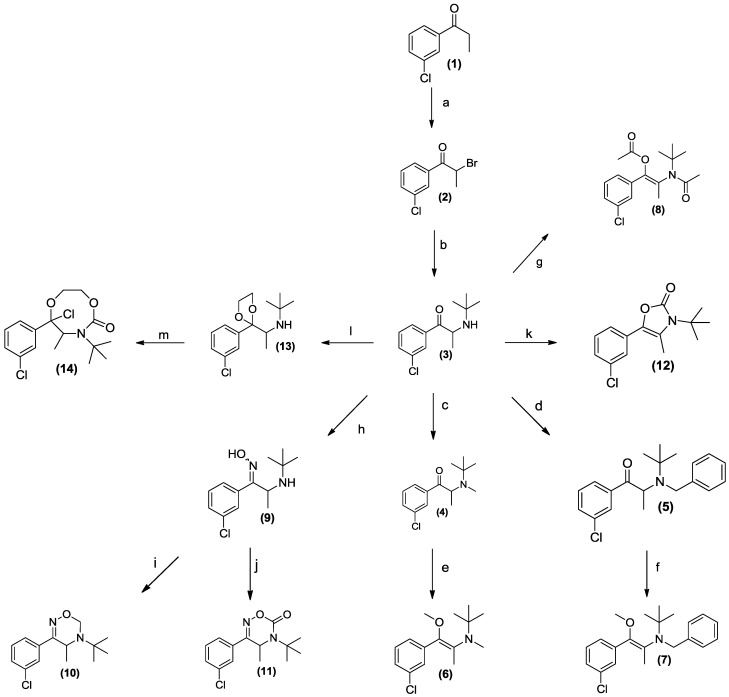
The synthesis of potential prodrug analogues of bupropion.

*Reagents and Conditions*: (**a**) DCM, Br_2_, RT, 30 min; (**b**) NMP, *t*-butylamine, 60 °C, 1 h; (**c**) ACN, DIPEA, CH_3_I, RT, 12 h; (**d**) ACN, DIPEA, BnBr, reflux, 36 h; (**e**) NaH, CH_3_I, DMF, RT, 30 min; (**f**) DMF, NaH, CH_3_I, RT, 2 h; (**g**) Ac_2_O, TEA, DMAP, −20 °C, 12 h; (**h**) Py, NH_2_OH.HCl, reflux, 2 h; (**i**) EtOH, *p*-TSA, paraformaldehyde, reflux, 8 h; (**j**) DCM, TEA, phosgene, 0 °C, 2 h; (**k**) ACN, TEA, phosgene, 0 °C, 2 h; (**l**) toluene, H_2_SO_4_, ethanediol, reflux, 60 h; (**m**) DCM, TEA, phosgene, 0 °C, 2 h.

The synthesis of the potential prodrugs of bupropion was carried out as depicted in Scheme 1 taking full advantage of its accessible functionality and readily available synthetic approaches. After characterization, prodrug candidates were evaluated using the *in vitro* screening matrix to predict processing following oral route of administration. The pharmacokinetics and metabolism were evaluated with the parent (bupropion) as a control. The *in vitro* pharmacological activity of the potential prodrug was also studied, which yielded interesting results. This type of approach to prodrug identification, involving systematic synthetic modification and assessment may be useful in other settings, most obviously with common phenylethylamino drugs.

## 2. Experimental Section

### 2.1. Instrumentation

Chromatographic analysis was carried out on a Thermo Accela LC (Thermo Fisher Scientific, Waltham, MA, USA). The detector was a Thermo LTQ-XL-Orbitrap Discovery mass spectrometer. Centrifugation was carried out on an IEC Micromax centrifugator (Thermo Fisher Scientific). Vortex mixing was carried out on a Velp Scientifica Rx3 vortex mixer (Velp Scientifica, Usmate, Italy). Standards were stored in a Thermo Forma −80 °C ULT freezer. Infra-red (IR) spectra were obtained using a Nicolet 205 FT Infra-red spectrometer (Thermo Fisher Scientific). Band positions are given in cm^−1^. Solid IR test samples were obtained as KBr discs; oils were analyzed as neat films on NaCl plates. ^1^H and ^13^C-NMR spectra were recorded at 27 °C on a Bruker DPX 400 MHz FT NMR spectrometer (400.13 MHz ^1^H, 100.61 MHz ^13^C) (Bruker, Billerica, MA, USA), in either CDCl_3_ or CD_3_OD. For CDCl_3_, ^1^H-NMR spectra were assigned relative to the CHCl_3_ 7.26 δ and ^13^C-NMR spectra were assigned relative to the middle CDCl_3_ triplet at 77.00 ppm. For CD_3_OD, ^1^H and ^13^C-NMR spectra were assigned relative to the centre peaks of CD_3_OD multiplets at 3.30 δ and 49.00 ppm respectively. Coupling constants are reported in Hertz. For ^1^H-NMR assignments, chemical shifts are reported: shift values (number of protons, description of absorption, coupling constant(s) where applicable, proton assignment). High resolution mass spectra were obtained on a Thermo LTQ XL-Orbitrap Discovery instrument. Flash column chromatography was carried out on Merck Silica Gel 60 (particle size 0.040–0.063 mm). Thin Layer Chromatography (TLC) was carried out on Merck Silica gel F254 plates (Merck KGaA, Darmstadt, Germany).

### 2.2. Materials

Bupropion hydrobromide bulk active ingredient was supplied from Biovail Technologies Ireland Ltd. (Dublin, Ireland). Pooled human liver microsomes, pooled human intestinal microsomes, guinea pig S9 fraction, and NADP regenerating solutions were supplied by BDBiosciences (Franklin Lakes, NJ, USA). Citrated human plasma was obtained from healthy male and female volunteers from Trinity College, Dublin. LC-MS grade solvents were purchased from Fisher Scientific Ireland (city, Ireland). Pepsin, from porcine stomach mucosa, with an activity of 800 to 2500 units per mg of protein (Sigma-Aldrich catalogue number P7000, St. Louis, MO, USA). Pancreatin (Sigma-Aldrich catalogue number P8096). Phosphate buffered saline tablets, (Sigma-Aldrich catalogue number P4417). HPLC grade solvents and all other reagents were analytical grade and purchased from Sigma Aldrich Ireland. Bupropion hydrochloride reference standard and its metabolite standards were purchased from Toronto Research Chemicals (North York, ON, Canada). All stock standards and solutions were sub-aliquotted and stored at −80 °C to reduce freeze thaw cycles.

### 2.3. Media Preparations

*Human liver microsome (HLM) stock solution preparation*. HLM (0.5 mL) stock was added to PBS solution (9.5 mL). Final concentration; 1 mg/mL. The PBS solution was prepared by addition of one USP PBS tablet (Sigma-Aldrich catalogue number P4417) dissolved in 200 mL deionised water.

*Human intestinal microsome (HIM) stock solution preparation*. HIM (0.2 mL) stock was added to PBS solution (9.8 mL). Final concentration; 0.2 mg/mL.

*Nicotinamide adenine dinucleotide phosphate (NADPH) regenerating solution A stock solution preparation*. NADPH solution A (5.0 mL) was added to PBS solution (5.0 mL). Final concentration; 13 mM NADP^+^.

*Nicotinamide adenine dinucleotide phosphate (NADPH) regenerating solution B stock solution preparation*. NADPH solution B (1.0 mL) was added to PBS (9.0 mL). Final concentration; 4 U/mL G-6-PDH.

*Pooled human plasma solution preparation*. Citrated human plasma was pooled together from three separate donors.

*USP simulated gastric fluid preparation*. Sodium chloride (0.02 g) and purified pepsin (0.032 g) that is derived from porcine stomach mucosa with an activity of 800 to 2,500 units per mg of protein were dissolved in 0.07 mL of concentrated hydrochloric acid and sufficient water to make 10 mL. This test solution had a pH of about 1.2. 

*USP simulated intestinal fluid preparation*. Monobasic potassium phosphate (0.068 g) was dissolved in 2.5 mL of water and mixed. Sodium hydroxide (0.77 mL of 0.2 M) and water (5.0 mL) were added. Pancreatin (0.1 g) was added and the pH adjusted with either 0.2 M sodium hydroxide or 0.2 M hydrochloric acid to a pH of 6.8 ± 0.1. The solution was diluted to 10 mL with water.

### 2.4. Chromatographic Method for Screening Potential Prodrugs

The column used for chromatographic separation was a Waters Xbridge C18, 2.1 × 50 mm 2.5 µm at 30 °C. Mobile phase A: 10:40:50, 1.0% ammonium hydroxide solution in water, adjusted to pH 10.5 with formic acid: water: acetonitrile. Mobile phase B: 10:90, 1.0% ammonium hydroxide solution in water, adjusted to pH 10.5 with formic acid: acetonitrile. The flow rate was 250 µL/min, injection volume: 10 µL, run time: 10 min. Gradient, 100% A hold for 1 min, 0% B to 100% B over 4 min, hold 100% B for 4.5 min, 0% B at 9.51 min and equilibrate for 0.5 min.

### 2.5. Mass Spectrometer Conditions for Detection of Potential Prodrugs

The LTQ-XL-Orbitrap mass spectrometer was coupled to the Accela LC system via an electrospray ionization (ESI) probe. The capillary temperature was maintained at 350 °C, sheath gas flow rate 50 arbitrary units, auxiliary gas flow rate 5 arbitrary units, sweep gas flow rate 0 arbitrary units, source voltage 3.20 kV, source current 100 µA, capillary voltage 43.00 V and tube lens 100 V. Compounds were detected in positive ion mode using selected ion monitoring (SIM). Hydroxybupropion was detected (M+H)^+^ = 256.1099, Retention time (RT) = 1.0 min, *Rac*-*erythro*-hydrobupropion (M+H)^+^ = 242.1306, RT = 2.3 min, *Rac*-*threo*-hydrobupropion (M+H)^+^ = 242.1306, RT 2.8 min, bupropion (M+H)^+^ = 240.1150, RT = 2.0 min. The optimum detector conditions were found by tuning the instrument to be most sensitive for bupropion’s most abundant ion at 240 (*m/z*).

### 2.6. Metworks Software

Metworks 1.1.0 software from Thermo Electron Corporation was used to screen accurate mass LC-MS chromatograms for phase I and phase II metabolic transitions. This was done in three steps. The sample (metabolized TIC/MS^n^) chromatogram and a blank chromatogram are selected from the sample run. The blank is subtracted from the sample to clean up the background spectra. The analyte(s) being metabolized are selected or inputted into the software, namely the exact mass molecular mass. Metabolite modifications are selected from the software or can be entered into the software manually. These modifications are the exact mass transitions that would be expected from the analyte of interest if the metabolic modification has taken place. The chromatogram is then screened by the software for each modification and the results are presented. The software increases productivity by automatically searching for metabolites of the analyte with exact mass specifications.

### 2.7. Determination of Microsomal Metabolic Stability

Microsomes, NADPH regenerating solutions A and B were thawed rapidly to 37 °C, then kept on wet ice until ready for use. A stock solution of the prodrug (5 mM) was prepared in ACN. To a 1.7 mL microcentrifuge tube in a 37 °C water bath was added the phosphate buffered saline solution (349 µL), the NADPH regenerating solution A (50 µL), the NADPH regenerating solution B (50 µL), and the prodrug solution (1 µL). After incubation for 5 min, microsome solution (50 µL) was added and the metabolism was initiated. The tube was vortex mixed. The final concentrations in this solution was 10 µM prodrug, 0.1 mg/mL HLM or 0.02 mg/mL HIM, 1.3 mM NADP^+^, 0.4 U/mL glucose-6-phosphate dehydrogenase and 0.2% organic solvent. This solution was incubated for 60 min at 37 °C. The reaction was quenched by addition of acetonitrile (500 µL). The mixture was centrifuged at 10,000 × *g* for 10 min. The supernatant was analyzed by LC-MS analysis.

### 2.8. Determination of Michaelis-Menten Kinetic Parameters

Microsomes, NADPH regenerating solutions A and B were thawed rapidly to 37 °C, and then kept on ice until ready for use. Substrate solutions were prepared (1–500 µM) where the maximum concentration of DMSO was 1%, and the concentration of microsomes, NADPH regenerating solution B and phosphate buffer was fixed. After incubation for 5 min, NADPH regenerating solution A was added and the metabolism was initiated. Final volume was 100 μL. The tube was inverted twice and vortex mixed. The final concentrations of prodrug in this solution was 1–500 µM, HLM (0.1 mg/mL) or HIM (0.02 mg/mL), NADP^+ ^ (1.3 mM), glucose-6-phosphate dehydrogenase (0.4 U/mL) and 1.0% DMSO. This solution was incubated for 20 min at 37 °C. The reaction was quenched by addition of 100 µL of acetonitrile. The mixture was centrifuged at 10,000 × *g* for 10 min. The supernatant was analyzed by LC-MS analysis.

### 2.9. Synthesis

*Synthesis of Rac-2-bromo-1-(3-chlorophenyl) propan-1-one* (**2**). To a stirred solution of **1** (10.0 g, 59.3 mmol) in dichloromethane (50 mL) in a round bottomed flask was added dropwise 1.0 M Br_2_ in dichloromethane until the bromine color remained. The residual dichloromethane and bromine was removed under vacuum to afford an oil. The oil was found to be pure by TLC analysis. (15.0 g, 99% yield).^1^H-NMR (CDCl_3_) δ (ppm): 1.90–1.92 (d, 3H, *J =* 6.71 Hz, -CH_3_), 5.22–5.27 (q, 1H, *J =* 6.6 Hz, -CH), 7.42–7.46 (t, 1H, *J =* 7.97 Hz, Ar-H), 7.56–7.58 (d, 1H, *J =* 8.04 Hz, Ar-H), 7.89–7.91 (d, 1H, *J =* 8.04 Hz, Ar-H), 7.99–8.00 (t, 1H, *J =* 1.75 Hz, Ar-H). ^13^C-NMR (CDCl_3_) δ (ppm): 19.5 (-CH_3_), 40.8 (-CH-Br), 126.5, 128.5, 129.6, 133.1, 134.6, 135.1 (6 × Ar-C), 191.6 (-C=O). IR υ_max_ (film) (cm^−1^): 1682 (-C=O). LRMS (M+H^+^); actual 246.95, found 247.1.

*Synthesis of Rac-bupropion hydrochloride* (**3**). To a stirred solution of **2** (15.0g, 60.6 mmol) in *N-*methyl-2-pyrrolidone (50 mL) in a round bottomed flask was added *t*-butylamine hydrochloride (6.64 g, 6.06 mmol). The mixture was heated to 60 °C for 60 min. The contents of the flask were then transferred to a separating funnel, 25 mL of water was added, and the mixture was extracted ether (3 × 25 mL). The combined ether extracts were washed water (3 × 25 mL) and dried over anhydrous magnesium sulphate. HCl gas was bubbled through the ether extracts providing the HCl salt. The excess ether was removed under vacuum and the solid remaining was washed with ether. After drying, the salt (**3**) was obtained as a white solid (16.6 g, 99% yield). ^1^H-NMR (CD_3_OD) δ (ppm): 1.16 (s, 9H, -C(CH_3_)_3_), 1.34–1.36 (d, 3H, *J =* 7.13 Hz, -CH_3_), 4.60–4.65 (q, 1H, *J =* 6.9 Hz, -CH), 7.62–8.14 (m, 4H, 4 × Ar-H). ^13^C-NMR (CD_3_OD) δ (ppm): 22.8 (-CH_3_), 30.1 (-C(CH_3_)_3_), 53.0 (-C(CH_3_)_3_), 53.9 (-CH), 128.6, 130.0, 132.3, 135.2, 136.9, 138.2 (6 × Ar-C), 204.6 (-C=O). IR υ_max_ (KBr) (cm^−1^): 2771 (NH), 1692 (-C=O). HRMS (M+H^+^); actual 240.1150, found 240.1147.

*Synthesis of Rac-N-methyl bupropion* (**4**). To a stirred solution of **3** (1.0 g, 3.1 mmol) in acetonitrile (25 mL) in a round bottomed flask was added *N*,*N-*diisopropylethylamine (0.8 g, 6.3 mmol) and methyl iodide (1.0 g, 7.1 mmol). The mixture was allowed to stir overnight at room temperature. An aqueous solution of saturated sodium bicarbonate (10 mL) was added. The mixture was extracted with ethyl acetate (3 × 10 mL). The ethyl acetate extracts were combined and dried over anhydrous magnesium sulphate. Silica gel (2 g) was added and the ethyl acetate removed under vacuum. This mixture was separated and purified into homogenous fractions by flash column chromatography using hexane:ethyl acetate (9:1) as mobile phase. The solvent was removed under vacuum to provide (**4**), as a pale yellow oil, 0.50 g (63%) yield. ^1^H-NMR (CDCl_3_) δ (ppm): 1.21 (s, 9H, -C(CH_3_)_3_), 1.27–1.28 (d, 3H, *J =* 6.55 Hz, -CHCH_3_), 2.18 (s, 3H, -NCH_3_), 4.59–4.64 (q, 1H, *J =* 7.0 Hz, -CHCH_3_), 7.36–8.09 (m, 4H, 4 × Ar-H). ^13^C-NMR (CDCl_3_) δ (ppm): 11.4 -CHCH_3_, 27.7 -C(CH_3_)_3_, 28.7 -NCH_3_, 54.7 -C(CH_3_)_3_, 57.0 -CHCH_3_, 126.2, 128.5, 128.9, 131.6, 133.8, 138.3 (6 x Ar-C), 200.7 -C=O. IR υ_max_ (film) (cm^−1^): 1701. HRMS (M+H^+^); actual 254.1306, found 254.1299.

*Synthesis of Rac-N-benzyl bupropion* (**5**). To a stirred solution of **3** (0.5 g, 1.6 mmol) in acetonitrile (5 mL) in a round bottomed flask was added *N*,*N-*diisopropylethylamine (0.60 g, 4.5 mmol) and benzyl bromide (0.29 g, 1.7 mmol). The mixture was refluxed for 36 h. The contents of the flask were washed into a separating funnel with water (2 × 5 mL), then ethyl acetate (10 mL). The mixture was washed with ethyl acetate (3 × 10 mL) and the aqueous layer was discarded. The organic layer was dried over anhydrous magnesium sulphate and reduced under vacuum. Silica gel (5 g) was added and the remaining solvent was removed under vacuum. This mixture was separated and purified into homogenous fractions by flash column chromatography using hexane-ethyl acetate (95:5) as mobile phase. The solvent was removed under vacuum to provide (**5**) as a bright yellow oil, yield 0.2 g (37.5%). ^1^H-NMR (CDCl_3_) δ (ppm): 1.20 (s, 9H, -C(CH_3_)_3_), 1.39–1.41 (d, 3H, *J =* 6.76 Hz, -CHCH_3_), 3.99–4.04 (d, 1H, *J =* 16.70 Hz, -CH_2_-), 4.10–4.14 (d, 1H, 16.68 Hz, -CH_2_-), 4.73–4.78 (q, 1H, *J =* 6.7 Hz, -CHCH_3_), 7.11–7.77 (m, 9H, 9 × Ar-H). ^13^C-NMR (CDCl_3_) δ (ppm): 13.9 -CH_3_, 28.8 -C(CH_3_)_3_, 47.7 -CHCH_3_, 55.9 -C(CH_3_)_3_, 56.2 -CH_2_-, 125.5 Ar-C, 125.9 Ar-C, 127.2 (2 × Ar-C), 127.4 (2 × Ar-C), 128.1 Ar-C, 128.9 Ar-C, 131.7 Ar-C, 133.9 Ar-C, 138.2 Ar-C, 142.5 Ar-C, 201.9 –C=O. IR υ_max_ (film) (cm^−1^): 1732. HRMS (M+H^+^); actual 330.1619, found 330.1611.

*Synthesis of E/Z-N,O-dimethylated bupropion* (**6**). To a stirred solution of **4** (0.05 g, 0.18 mmol) in dry DMF (3 mL) in a 10 mL round bottomed flask was added sodium hydride (60% in mineral oil, 0.01 g, 0.4 mmol). The solution was left stirring at room temperature for 30 min under a nitrogen atmosphere. Methyl iodide (0.03 g, 0.2 mmol) was added drop wise. After 2 h the reaction was quenched by addition of a saturated aqueous solution of sodium bicarbonate (10 mL). The mixture was extracted with diethyl ether (3 × 5 mL). The diethyl ether extracts were combined and dried over anhydrous magnesium sulphate. Silica gel (1 g) was added and the diethyl ether removed under vacuum. This mixture was separated and purified into homogenous fractions by flash column chromatography using hexane:ethyl acetate (9:1) as mobile phase. The solvent was removed under vacuum to provide (**6**) as a yellow oil, 0.02 g (42%). ^1^H-NMR (CDCl_3_) δ (ppm): 0.96 (s, 9H, -C(CH_3_)_3_), 1.85 (s, 3H, -CH_3_), 2.49 (s, 3H, -NCH_3_), 3.40 (s, 3H, -OCH_3_), 7.20–7.70 (m, 4H, 4 × Ar-H). ^13^C-NMR (CDCl_3_) δ (ppm): 12.0 -CH_3_, 27.3 -C(CH_3_)_3_, 33.6 -NCH_3_, 54.8 -C(CH_3_)_3_, 57.2 -OCH_3_, 126.2 (2 × Ar-C), 128.2 Ar-C, 128.5 Ar-C, 132.7, 133.7 (2 × Ar-C), 137.1, 150.3 (2 × –C=C-). IR υ_max_ (film) (cm^−1^): 1621, 1211. HRMS (M+H^+^); actual 268.1463, found 268.1461.

*Synthesis of (E/Z)-O-methyl N-benzyl bupropion* (**7**). To a stirred solution of **5** (0.1 g, 0.33 mmol) in dry DMF (3 mL) at room temperature in a round bottomed flask was added sodium hydride (0.01 g, 0.4 mmol). The solution was allowed to stir at room temperature for 30 min. Methyl iodide (0.05 g, 0.35 mmol) was added slowly and left stirring at room temperature for 2 h. A saturated aqueous solution of sodium bicarbonate was added (10 mL) and the mixture was quantitatively transferred to a separating funnel. The mixture was extracted with hexane (3 × 10 mL). The hexane extracts were combined and dried over anhydrous magnesium sulphate. Silica gel (1.0 g) was added and the solvent removed under vacuum. The mixture was transferred onto a flash column and purified into homogenous fractions by flash column chromatography using hexane-ethyl acetate (50:1) as mobile phase. The solvent was removed under vacuum. After drying, a yellow oil remained (**7**, one isomer), yield 0.02g (15%). ^1^H-NMR (CDCl_3_) δ (ppm): 1.20 (s, 9H, -C(CH_3_)_3_), 1.99 (s, 3H, -CH_3_), 3.21 (s, 3H, -OCH_3_), 3.66–3.70 (d, 1H, *J =* 12.9 Hz, -CH_2_-), 3.93–3.96 (d, 1H, *J =* 12.8 Hz, -CH_2_-), 7.00–7.75 (m, 9H, 9 × Ar-H). ^13^C-NMR (CDCl_3_) δ (ppm): 12.0, 28.0, 29.3, 49.5, 55.8, 57.0, 126.0, 126.1, 127.1, 127.6, 128.5, 129.3, 129.7, 132.3, 136.5, 138.7. IR υ_max_ (film) (cm^−1^): 1610, 1016. HRMS (M+H^+^); actual 344.1776, found 344.1785.

*Synthesis of the (E/Z)-N,O-diacetylated bupropion* (**8**). To a stirred solution of **3** (0.5g, 1.6 mmol) in acetic anhydride (5 mL) at −20 °C in a round bottomed flask (10 mL) was added triethylamine (0.5 g, 5 mmol) and a catalytic amount of *N*,*N-*dimethylaminopyridine. The temperature was allowed to gradually rise to room temperature with constant stirring overnight. The reaction was quenched by the slow, drop wise addition of a saturated aqueous sodium bicarbonate until the effervescence of carbon dioxide ceased and a precipitate remained. The contents were transferred to a separating funnel and the mixture was extracted with hexane (3 × 20 mL). The organic layers were combined, dried over anhydrous magnesium sulphate and filtered. Silica gel (5.0 g) was added to the filtrate and the solvent removed under vacuum. This mixture was separated and purified into homogenous fractions by flash column chromatography using hexane-ethyl acetate (9:1) as mobile phase. The solvent was removed under vacuum. The combined *E* and *Z* isomers were isolated as a clear oil, yield 0.19 g (37.1%). ^1^H-NMR (CDCl_3_) δ (ppm): 1.51–1.52 (m, 9H, -C(CH_3_)_3_), 1.96–1.97(m, 3H, -CH_3_), 2.12–2.15 (m, 6H, 2 × C=OCH_3_), 7.29–7.38 (m, 4H, 4 × Ar-H). ^13^C-NMR (CDCl_3_) δ (ppm): 20.4 (-CC=OCH_3_), 20.6 (-CC=OCH_3_), 23.7 -CH_3_, 28.0 -C(CH_3_)_3_, 58.0 -C(CH_3_)_3_, 126.3, 127.9, 128.7, 129.3 (4 × Ar-C), 128.2, 133.9, 135.4, 143.9 (4 × Ar-C), 167.8 -C=O, 169.5 -C=O. IR υ_max_ (film) (cm^−1^): 1742, 1685. HRMS (M+H^+^); actual 324.1361, found 324.1357.

*Synthesis of syn- and anti-bupropion hydroxyimine* (**9**). To a round bottomed flask (50 mL) was added a solution of **3** (1.0 g, 3.12 mmol) in pyridine (10 mL) and hydroxylamine hydrochloride (0.28 g, 4.0 mmol). The solution was refluxed under constant stirring for 2 h. The solvent was removed from the reaction following the sequential addition of toluene (3 × 25 mL) and removal of the solvent under vacuum after each addition. To the resulting residue was added ethyl acetate (10 mL) and then silica gel (5.0 g) and the mixture was dried under vacuum and directly loaded onto a flash column. The product from the reaction was eluted with dichloromethane-methanol (9:1) as mobile phase to provide the syn and anti-forms of the oxime (**9**) as a white solid, yield 0.2 g (37.5%). ^1^H-NMR (CDCl_3_) δ (ppm): 1.21 (s, 9H, -C(CH_3_)_3_), 1.26–1.28 (d, 3H, *J =* 6.87 Hz, -CHCH_3_), 3.82–3.87 (q, 1H, *J =* 6.8 Hz, -CHCH_3_), 7.29–7.47 (m, 4H, 4 × Ar-H). ^13^C-NMR (CDCl_3_) δ (ppm): 22.9 -CHCH_3_, 29.1 -C(CH_3_)_3_, 51.7 -CHCH_3_, 52.2 -C(CH_3_)_3_, 126.4, 128.3, 128.9, 129.5, 134.2, 134.5 (6 × Ar-C), 157.6 -C=N-. M.p.: 202–206 °C. IR υ_max_ (KBr) (cm^−1^): 3434, 3301, 1651. HRMS (M+H^+^); actual 255.1259, found 255.1249.

*Synthesis of* *Rac-oxadiazine of bupropion* (**10**). To a stirred solution of **9** (0.3 g, 1.2 mmol) in ethanol (10 mL) in a 50 mL round bottomed flask was added paraformaldehyde (1.1 g) and a catalytic amount of *p*-toluenesulfonic acid. This mixture was refluxed for 8 h, after which an aqueous saturated sodium bicarbonate solution (10 mL) was added and the mixture was transferred to a separating funnel. It was then extracted with ethyl acetate (3 × 10 mL). The ethyl acetate extracts were combined and dried over anhydrous magnesium sulphate. Silica gel (1 g) was added and the ethyl acetate removed under vacuum. This mixture was separated and purified into homogenous fractions by flash column chromatography using hexane-ethyl acetate (9:1) as mobile phase. The solvent was removed under vacuum to yield (**10**) as a white solid (0.1g, 31%). ^1^H-NMR (CDCl_3_) δ (ppm): 1.26 (s, 9H, -C(CH_3_)_3_), 1.30–1.31 (d, 3H, *J =* 7.00 Hz, -CH_3_), 3.90–3.95 (q, 1H, *J =* 6.8 Hz, -CHCH_3_), 4.74–4.77 (d, 1H, *J =* 11.23 Hz, -CH_2_), 5.14–5.17(dd, 1H, *J =* 1.81 and 9.88 Hz, -CH_2_), 7.34–7.57 (m, 4H, 4 × Ar-H). ^13^C-NMR ppm: 21.3 -CHCH_3_, 28.2 -C(CH_3_)_3_, 44.1 -CHCH_3_, 55.0 -C(CH_3_)_3_, 75.3 -CH_2_, 124.2, 126.2, 129.5, 129.9, 134.7, 137.3 (6 × Ar-C), 160.4 -C=N-. M.p.: 169–175 °C. IR υ_max_ (KBr) (cm^−1^): 1673. HRMS (M+H^+^); actual 267.1259, found 267.1250.

*Synthesis of* *Rac-oxadiazinone of bupropion* (**11**). To a stirred solution of **9** (0.5 g, 2.0 mmol) in dry dichloromethane (30 mL) in a round bottomed flask under a nitrogen atmosphere was added triethylamine (0.7 g, 7.1 mmol). The mixture was chilled to 0 °C and phosgene (a 20% solution in toluene, 2.0 g, 4 mmol) was added dropwise. The mixture was allowed to equilibrate to room temperature over 2 h. A gentle stream of nitrogen gas was passed through the mixture to remove any residual phosgene. An aqueous solution of saturated sodium bicarbonate (10 mL) was added slowly. The mixture was extracted with diethyl ether (3 × 10 mL). The diethyl ether extracts were combined and dried over anhydrous magnesium sulphate. Silica gel (1 g) was added and the diethyl ether removed under vacuum. This mixture was separated and purified into homogenous fractions by flash column chromatography using initially 100% hexane, but with an increasing gradient of ethyl acetate as mobile phase, to 100% ethyl acetate. The solvent was removed under vacuum to give **11** as a yellow oil, 0.1 g (18%) yield. ^1^H-NMR (CDCl_3_) δ (ppm): 1.45–1.47 (d, 3H, *J =* 6.89 Hz, -CH_3_), 1.57 (s, 9H, -C(CH_3_)_3_), 4.73–4.78 (q, 1H, *J =* 6.8 Hz, -CHCH_3_), 7.44–7.72 (m, 4H, 4 × Ar-H). ^13^C-NMR (CDCl_3_) δ (ppm): 18.4 -CH_3_, 28.2 -C(CH_3_)_3_, 47.2 -CHCH_3_, 57.4 -C(CH_3_)_3_, 124.0, 126.0, 130.0, 131.0, 131.2, 134.9 (6 × Ar-C), 151.3 -C=N-, 160.3 -C=O. IR υ_max_ (film) (cm^−1^): 1692, 1641. HRMS (M+H^+^); actual 281.1051, found 281.1040.

*Synthesis of the oxazolone of bupropion* (**12**). To a stirred solution of **3** (1.0 g, 3.1 mmol) in dry dichloromethane (30 mL) in a 50 mL round bottomed flask under a nitrogen atmosphere was added triethylamine (1.7 g, 12.5 mmol). The mixture was chilled to 0 °C and phosgene (20% in toluene, 10 g, 20 mmol) was added dropwise. The mixture was allowed to equilibrate to room temperature over 2 h. A gentle stream of nitrogen gas was passed through the mixture to remove any residual phosgene. An aqueous solution of saturated sodium bicarbonate (10 mL) was added slowly. The mixture was extracted with diethyl ether (3 × 10 mL). The diethyl ether extracts were combined and dried over anhydrous magnesium sulphate. Silica gel (1 g) was added and the diethyl ether removed under vacuum. This mixture was separated and purified into homogenous fractions by flash column chromatography using initially 100% hexane, but with an increasing gradient of ethyl acetate as mobile phase to 100% ethyl acetate. The solvent was removed under vacuum to give **12** as a white solid, 0.60 g (73%) yield. ^1^H-NMR (CDCl_3_) δ (ppm): 1.60 (s, 9H, -C(CH_3_)_3_), 2.30 (s, 3H, -CH_3_), 7.14–7.34 (m, 4H, 4 × Ar-H). ^13^C-NMR (CDCl_3_) δ (ppm): 13.2 -CH_3_, 29.0 -C(CH_3_)_3_, 58.0 -C(CH_3_)_3_, 120.7 (-C=C-), 123.9 (Ar-C), 125.7 (Ar-C), 127.1 (Ar-C), 129.4 (Ar-C), 129.5 (-C=C-), 133.0 (Ar-C), 133.9 (Ar-C), 153.5 (-C=O). M.p.: 178–180 °C. IR υ_max_ (KBr) (cm^−1^): 1645 (-C=O), 1152 (-CO-). LRMS (M+H^+^); actual 266.09, found 266.1.

*Synthesis of a Rac-dioxolane of bupropion* (**13**). To a stirred solution of **3** (1.0 g, 3.1 mmol) in toluene (50 mL) in a double neck round bottomed flask (250 mL) was added 1,2-ethanediol (2.0 mL, 32 mmol) and concentrated sulfuric acid (0.1 mL, 1.0 mmol). A Dean-Stark apparatus was attached to the vertical neck of the flask and the mixture was refluxed for 60 h at 200 °C. The flask was replenished with ethanediol and toluene required during the water removal process. After completion, an aqueous solution of saturated sodium bicarbonate (10 mL) was added. The mixture was extracted with ethyl acetate (3 × 10 mL). The ethyl acetate washings were combined and dried over anhydrous magnesium sulphate. The ethyl acetate was removed under vacuum to afford a residue which required the unreacted ketone to be reduced before the co-eluting mixture could be purified. This was achieved by dissolving the mixture in methanol (10 mL) and treating the solution with sodium borohydride (0.1 g, 2.6 mmol). An aqueous solution of saturated sodium bicarbonate (10 mL) was added. The mixture was extracted with ethyl acetate (3 × 10 mL). The ethyl acetate extracts were combined and dried over anhydrous magnesium sulphate. Silica gel (1 g) was added and the ethyl acetate removed under vacuum. This mixture was separated and purified into homogenous fractions by flash column chromatography using hexane-ethyl acetate (9:1) as mobile phase. The solvent was removed under vacuum to provide (**13**) as a clear oil, 0.6 g (68%) yield. ^1^H-NMR (CDCl_3_) δ (ppm): 0.93 (s, 9H, -(CH_3_)_3_), 1.06–1.08 (d, 3H, *J =* 6.56 Hz, -CH_3_), 2.99–3.04 (q, 1H, *J =* 6.5 Hz, -CH), 3.72–3.83 (m, 2H, -CH_2_-), 3.40–4.10 (m, 2H, -CH_2_-), 7.25–7.50 (m, 4H, 4 × Ar-H). ^13^C-NMR (CDCl_3_) δ (ppm): 18.7 (-CH_3_), 29.4 -C(CH_3_)_3_, 50.1 -C(CH_3_)_3_, 53.4 -CH-, 64.3 -CH_2_-, 64.5 -CH_2_-, 110.7 -CO_2_-, 124.8, 126.9, 127.3, 128.4, 133.1, 143.5 (6 × Ar-C). IR υ_max_ (film) (cm^−1^): 3462 (NH), 1175 (-CO_2_-). LRMS (M+H^+^); actual 284.14, found 284.1.

*Synthesis of a (Rac)-8-membered ring cyclic carbamate of bupropion* (**14**). To a stirred solution of **13** (0.3 g, 1.0 mmol) in dry dichloromethane (5 mL) in a 10 mL round bottomed flask under a nitrogen atmosphere was added triethylamine (0.3 g, 3.0 mmol). The mixture was chilled to 0 °C and phosgene (20% in toluene, 1.0 g, 2 mmol) was added dropwise. The mixture was allowed to equilibrate to room temperature over 2 h. A gentle stream of nitrogen gas was passed through the mixture to remove any residual phosgene. An aqueous solution of saturated sodium bicarbonate (10 mL) was added slowly. The mixture was extracted with diethyl ether (3 × 10 mL) and analysis by TLC indicated the presence of two compounds. The diethyl ether washings were combined and dried over anhydrous magnesium sulphate. Silica gel (1 g) was added and the solvent removed under vacuum. This mixture was separated and purified into homogenous fractions by flash column chromatography using initially 100% hexane grading to hexane-ethyl acetate (1:1) as mobile phase. After removal of the solvent from the respective homogenous fractions the diastereomeric products were obtained as a colorless oil 0.12 g (35%) yield, and a yellow oil 0.07 g (20%). Diastereomer 1; ^1^H-NMR (CDCl_3_) δ (ppm): 0.79 (d, 3H, *J =* 6.61 Hz, -CH_3_), 1.48 (s, 9H, -C(CH_3_)_3_), 3.38–3.45 (m, 1H, -CH_2_-), 3.56–3.59 (m, 2H, -CH_2_-), 3.75–3.82 (m, 1H, -CH_2_-), 3.96–4.01 (q, 1H, *J =* 6.6 Hz, -CH-), 7.37–7.52 (m, 4H, 4 × Ar-H). ^13^C-NMR (CDCl_3_) δ (ppm): 19.3 -CH_3_, 28.2 -C(CH_3_)_3_, 43.1 -CH_2_-, 54.1 -C(CH_3_)_3_-, 61.4 -CH-, 63.6 -CH_2_-, 105.4 -COCl-, 123.9, 125.9, 129.6, 130.0, 134.9, 136.6 (6 × Ar-C), 153.8 (-C=O). IR υ_max_ (KBr) (cm^−1^): 3330, 1729, 1263. LRMS (M+H^+^); actual 346.09, found 346.2. Diastereomer 2; ^1^H-NMR (CDCl_3_) δ (ppm): 0.80–0.82 (d, 3H, *J =* 6.61 Hz, -CH_3_), 1.50 (s, 9H, -C(CH_3_)_3_), 3.40–3.46 (m, 1H, -CH_2_-), 3.59–3.62 (m, 2H, -CH_2_-), 3.90–3.86 (q, 1H, *J =* 6.6 Hz, -CH_2_-), 3.99–4.05 (m, 1H, -CH-), 7.30–7.61 (m, 4H, 4 × Ar-H). ^13^C-NMR (CDCl_3_) δ (ppm): 19.3 -CH_3_, 28.2 -C(CH_3_)_3_, 43.1 -CH_2_-, 54.1 -C(CH_3_)_3_-, 61.4 -CH-, 63.6 -CH_2_-, 105.4 -COCl-, 123.9, 125.9, 129.6, 130.0, 134.6, 136.6 (6 × Ar-C), 153.8 (-C=O). IR υ_max_ (film) (cm^−1^): 3333, 1735, 1251. LRMS (M+H^+^); actual 346.09, found 346.1.

### 2.10. LCMS Method Validation

The experimental of the analytical method validation can be found in the Supplemental Information.

### 2.11. Neuropharmacology of N-Methyl Bupropion and Bupropion

The experimental of the pharmacological studies can be found in the Supplemental Information.

## 3. Results and Discussion

### 3.1. Chemistry

We have previously reported that bupropion is susceptible to base catalyzed hydrolysis and oxidation to four main degradation products under aqueous conditions [26]. In our hands methods of prodrug synthesis that utilize non*-*aqueous alkaline conditions also caused extensive degradation especially at or above the p*Ka* of bupropion and at elevated temperatures. For example the enamine form of bupropion may tautomerize to the imine form promoting hydrolysis to the corresponding α-hydroxyketone derivative, Scheme 1.

A possible solution to its instability is to prepare suitable prodrug forms of bupropion and in doing so exploit in particular the properties of its keto and amino functionalities. Although the keto group can undergo reactions typical of aldehydes and ketones, the neighboring amino group and chlorine in the *meta*-position on the aromatic ring have an effect on the reactivity of the keto carbon due to their electronic properties [27]. The *t*-butyl substituent on the amino group renders it less nucleophilic than would be expected of a typical secondary amine due to steric factors-it therefore does not readily undergo some reactions typical of secondary amines. Another factor depressing the nucleophilicity of the amine group in bupropion is the neighboring carbonyl group.

**Scheme 2:**
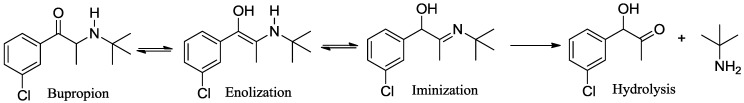
Keto-enol/enamine-imine tautomerism and hydrolysis of bupropion.

Numerous carbamate and amido derivative prodrugs of amines have been reported because of the ubiquity of relevant mammalian hydrolases [28]. In our studies with bupropion, we considered these kinds of approaches as well as alkylation using methyl iodide and benzyl bromide. Our rationale for forming alkylated derivatives was centered on increasing bupropion’s lipophilic character with the ultimate aim of increasing the permeability of the prodrug across the blood brain barrier. Enzyme types such as flavin containing mono-oxygenases (FMO’s) and cytochrome P450 oxidases, which are known to be also present in brain tissue, have the ability to dealkylate tertiary lipophilic *N-*alkylated amines to the more hydrophilic secondary or primary parent amine. Conversion of the secondary amine group in bupropion to a tertiary amine could also stabilize the molecule from degradation by removing the ability of imination as shown in Scheme 2.

Our strategy with the keto-group was to initially prepare its oxime and imine derivatives as these are functional groups known to be activated by both non*-*enzymatic mechanisms as well as by endogenous enzymes. For example, buparvaquone hydroxyimine [29] and nabumetone hydroxyimine [30] are both readily oxidized by CYP3A4 back to the parent ketone. A second objective was to enolise the keto group and subsequently form enol ethers and enol esters as these functional groups have been shown to be susceptible to enzymatic systems *in vivo* [31].

Bupropion is also an attractive compound from the multistep prodrug standpoint as it serves as a model for the presentation of a double prodrug, containing two potential prodrug functional groups. Examples of these forms include ximelagatran [32,33], a double prodrug of the anticoagulant drug melagatran, which is dealkylated and dehydroxylated mainly in the liver and acts as a direct thrombin inhibitor. In principle there are many reasons to consider this approach with bupropion as the double prodrug may have higher lipophilicity and should traverse membranes such as the intestinal mucosa and blood brain barrier more efficiently than a less lipophilic drug.

Some secondary and tertiary alkyl amines are reported to undergo dealkylation mediated by MAO-B to an amine and the corresponding aldehyde or ketone [34]. Accordingly, bupropion (**3**) was *N-*methylated with methyl iodide in the presence of diisopropylethylamine and acetonitrile at room temperature to form (**4**). Treatment of (**4**) with HCl gas in diethyl ether afforded the hydrochloride salt. Bupropion (**3**) was benzylated to (**5**) by treatment of the amine with benzyl bromide in diisopropylethylamine in acetonitrile.

Treatment of *N-*methyl bupropion (**4**) with sodium hydride in DMF resulted in formation of the sodium enolate of *N-*methyl bupropion. The enolate was treated with a methylating reagent usually methyl iodide or dimethyl sulfate. Both approaches resulted in the successful synthesis of the potential double prodrug of bupropion (**6**). *N-*benzyl bupropion (**5**) was converted to the *O*-methylenol ether analogue (**7**) in a similar fashion by treatment with methyl iodide or dimethyl sulfate in the presence of NaH.

While in principle one might anticipate that treatment of bupropion (**3**) with an acylation reagent might only furnish the corresponding amide derivative, in our situation the most appropriate route to a double prodrug of (**3**) was by its treatment with the corresponding anhydride in the presence of a tertiary base. The approach used involved treatment of (**3**) with Ac_2_O, DMAP and TEA in acetonitrile at room temperature. This furnished the (**8**) in poor yield which was optimized by simply using Ac_2_O as solvent and by conducting the reaction at <0 °C.

Bupropion (**3**) was converted to the hydroxyimine (**9**) by treatment with hydroxylamine hydrochloride in pyridine at ambient conditions. Having succeeded with the synthesis of oxime (**9**), it was felt that treatment of this oxime with formaldehyde and a catalytic amount of *p*-toluenesulfonic acid, one could bridge the amine and hydroxyimine with a methylene group to form (**10**), a potential double prodrug of bupropion. Treatment of the hydroxyimine (**9**) with paraformaldehyde in ethanol using *p*-toluenesulfonic acid under reflux yielded the oxadiazine (**10**).

As oxodioxolones are readily hydrolysed back to the parent drug [35], we envisaged in an analogous fashion that perhaps the oxadiazinone derivative (**11**) of bupropion may also be converted back to bupropion *in vivo*. Treatment of the hydroxyimine (**9**) with phosgene in dry dichloromethane and triethylamine gave the ring closed oxadiazinone (**11**).

As oxazolidine prodrugs of ephedrine have been synthesized [36,37], we hypothesized that using the method akin to that which resulted in the formation of (**11**), that it might be possible to furnish the oxazolone (**12**). Treatment of bupropion (**3**) with phosgene in acetonitrile with triethylamine base at low temperature readily afforded the cyclic oxazolone (**12**). Indeed this compound has been synthesized previously by Hamad *et al*. [24] for a naltrexol codrug application.

Carbamates are generally easily prepared by treatment of the primary or secondary amine with the corresponding chloroformate in the presence of a tertiary base [38]. Attempts were made to furnish carbamate derivatives of bupropion (**3**) by treating it with methyl and benzyl chloroformates in the presence of diisopropylethylamine, *N*,*N-*dimethylaminopyridine as catalyst in dry DCM at room temperature as well as by conducting the reaction at elevated temperatures or by using microwave conditions but all were unsuccessful. The reagent di-*tert*-butyl-dicarbonate was also used under similar conventional and microwave conditions but again there was no reaction. The inability of the amino group of bupropion to react with either chloroformates or di-*tert*-butyl-dicarbonate may be attributed to the reduced nucleophilicity and steric issues with the *t*-butyl group.

In an effort to increase the reactivity of the amine group in bupropion, the neighboring carbonyl was converted to the ketal (**13**). The reaction was optimized under Dean Stark conditions using sulfuric acid as catalyst, toluene and ethylene glycol. Treatment of (**13**) under the conditions described above with bupropion failed to secure any of the desired carbamates, suggesting that steric issues is likely to the central reason for the inactivity of the amino group.

Our focus then turned to a two-step procedure to secure the carbamate based prodrugs. The first step used phosgene to form a chloride derivative and then it was hypothesized that treatment of this intermediate with a variety of alcohols should in principle secure the desired carbamates. However treatment of the ketal (**13**) with phosgene in dichloromethane using triethylamine as base under a nitrogen atmosphere at 0 °C afforded the cyclic carbamate (**14**) as a mixture of stereoisomers as evidenced by TLC, HPLC and NMR analysis of the purified diastereoisomeric products. Although (**14**) was not an obvious choice as a bupropion prodrug, it was an interesting substance nevertheless for this application, as possible release of the carbamate group followed by dealkylation and loss of HCl would be expected to give the enol which upon tautomerisation should furnish bupropion.

### 3.2. Development of a Screening Tool to Evaluate Drug Metabolic Stability

**Figure 2:**
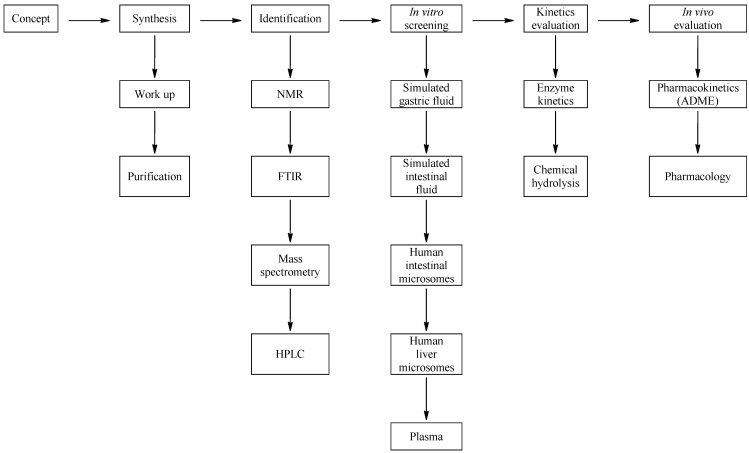
Schematic of the approach used for drug evaluation.

A summary of the oral prodrug candidate matrix we developed is illustrated in Figure 2. It contained six main conditions and can be applied to the development of any drug candidate. The work plan starts out with a concept molecule which in this case was bupropion. At this stage prodrug candidates are identified and the literature is searched for transformations that have been reported and methods used to carry out these transformations. This gave a background into the novelty of the prodrug concept for bupropion and allowed preparation for the synthetic approach. After synthesis and purification of each potential prodrug, full spectroscopic characterization was performed. Each prodrug was screened in the metabolic media to assess its stability. Potential prodrug candidates were then selected for further *in vitro* enzyme kinetic and hydrolysis studies to evaluate the most likely prodrug for further *in vivo* assessment.

### 3.3. Media Selection

The first obstacle prodrugs of bupropion are expected to encounter is the stomach which exposes candidates to low pH and gastric enzymes, mainly pepsin. Pepsin is most efficient at cleaving peptide bonds between hydrophobic and aromatic amino acids such as phenylalanine, tryptophan and tyrosine. In order to simulate the pH and digestive enzymes of the stomach, USP simulated gastric fluid (SGF) was used. Conditions in the intestine were simulated using USP simulated intestinal fluid (SIF).

The intestinal cells contain cytochome P450 enzymes, flavin monoxygenases, esterases and UDP glucuroyl transferases. Condition III involved the use of pooled human intestinal microsomes (HIM) in order to simulate the potential metabolism that occurs during absorption and passage through the intestinal wall.

After passing through the intestinal membrane into the portal circulation the prodrug quickly enters the liver. This metabolic condition was simulated in the present work by using pooled human liver microsomes (HLM).

Finally, pooled human plasma (PHP) was used to evaluate the prodrug stability/activation in blood since the plasma fraction contains much of the hydrolase activity in blood.

The five media (SGF, SIF, HIM, HLM and PHP) best represent the transit a drug or prodrug would take if delivered by the oral route of administration.

### 3.4. Chromatographic Methodology

A high pH reverse phase gradient method was optimized to chromatograph the prodrug and anticipated metabolite mixtures. This was coupled to high resolution MS detection. Although the mass spectrometer could separate out the masses of the individual compounds, the chromatographic method was still important as diastereomers, namely the bupropion amino alcohols, had the exact same mass and therefore needed to be separated chromatographically in order to be adequately identified and quantified.

### 3.5. Mass Spectrometric Method

To achieve maximum sensitivity of both known and unknown metabolites with the Orbitrap mass spectrometric detector, the system was set up with two scan types. The first scan was selective ion monitoring, scanning specifically for the accurate mass molecular ions of bupropion and its metabolites. The second scan type was full scan total ion chromatogram. This was used to obtain a mass spectrum of each unknown coming into the detector. Appended to this scan, the instrument was set up in data dependent acquisition mode. When the instrument detected a mass ion with a chlorine isotope pattern, this triggered a MS/MS and that ion was sent into the ion trap for MS^3^ analysis. This greatly improved the selectivity of the instrument for MS^n^ analysis as instantly only compounds relating to bupropion could be selected out due to their inclusion of the chlorine isotope.

### 3.6. LCMS Method Validation

The results of the analytical method validation can be found in supplemental information.

### 3.7. Screening Results

Table 1 summarizes the results obtained of each prodrug candidate following incubation in the selected media at 37 °C for one hour. One hour metabolism or hydrolysis was considered to be sufficient time to generate detectable levels of metabolites, bearing in mind the likely exposure time *in vivo*. A blank media was used in each analysis to subtract a background from each LC-MS sample chromatogram. A *t*_0_ sample was also used to identify impurities related to each prodrug.

After one hour incubation of each compound in the selected media, the mixture was quenched with acetonitrile and the sample was centrifuged to remove precipitated proteins. The supernatant solution was analyzed immediately by LC-MS using data dependent acquisition analysis. This data was then screened using Metworks software in order to establish what metabolites were present and more importantly, if bupropion or its metabolites were present. Bupropion and its metabolite standards were run with every prodrug analysis. A linear curve generated for bupropion and its metabolites was the determining factor if the system was suitable for analysis. The linear range for bupropion and metabolites was 1–250 ng/mL, r^2^ ≥ 0.9950.

**Table 1:** The testing matrix of potential prodrugs and hydrolysis/metabolism results. LOD = Limit of detection.

Prodrug tested	Primary product detected in media				
	SGF	SIF	HIM	HLM	PHP
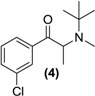	<LOD	<LOD	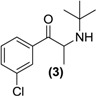	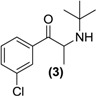	<LOD
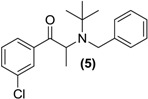	<LOD	<LOD	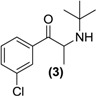	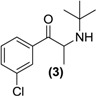	<LOD
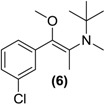	<LOD	<LOD	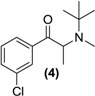	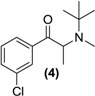	<LOD
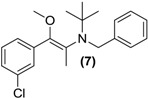	<LOD	<LOD	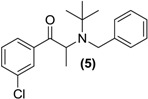	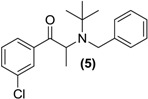	<LOD
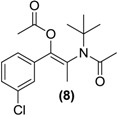	<LOD	<LOD	<LOD	<LOD	<LOD
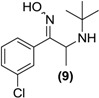	<LOD	<LOD	<LOD	<LOD	<LOD
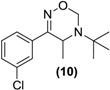	<LOD	<LOD	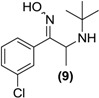	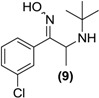	<LOD
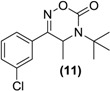	<LOD	<LOD	<LOD	<LOD	<LOD
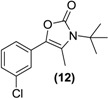	<LOD	<LOD	<LOD	<LOD	<LOD
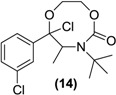	<LOD	<LOD	<LOD	<LOD	<LOD

For microsome solutions (HIM and HLM), the velocity of bupropion hydroxylation to hydroxybupropion was measured. This transformation is commonly used to assess activity of oxidative enzyme CYP2B6 in human liver and intestinal microsomes. Results reported as <LOD (less than limit of detection), indicated that bupropion, its metabolites or any of the other prodrugs were not detected in the media after a one hour incubation at 37 °C (*n =* 2). The limit of detection was 0.1 ng/mL for bupropion and expected metabolites. It was important to report if any of the other prodrugs were detected if for instance a possible multistep-prodrug was found. It was also important to report the metabolites of bupropion if for instance the prodrug was converted to bupropion but then subsequently metabolized to one of its metabolites immediately.

**Figure 3:**
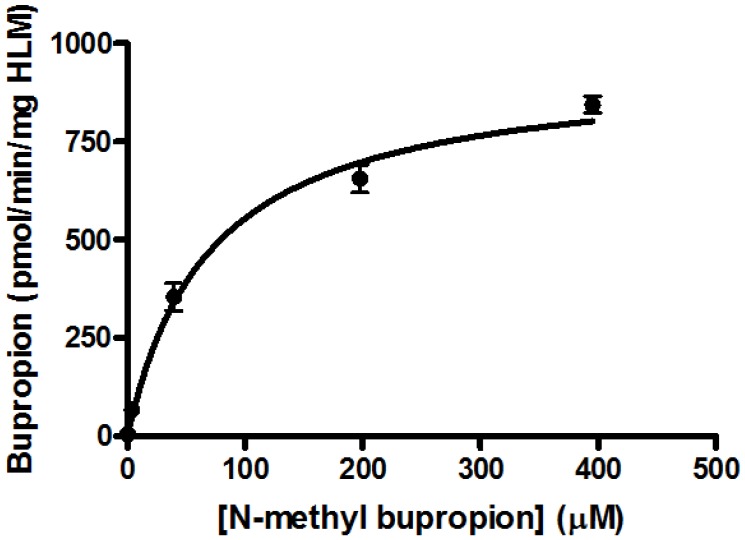
Substrate saturation study in pooled human liver microsomes of *N-*methylbupropion prodrug, (**4**). V_max_ = 900.4 ± 73.8 pmol/min/mg HLM, K_m_ = 96.8 ± 24.4 μM, R^2^ = 0.9289, *n =* 6.

*N-*methylbupropion (**4**) was a good candidate as a potential prodrug of bupropion. *N-*dealkylation is a common bio-transformation for xenobiotics *in vivo*, and oxidative enzymes have been shown to dealkylate quite sterically hindered groups. It was incubated in all five media and as anticipated, bupropion was detected in both the human liver and human intestinal microsome media. Further deconvolution of the data by Metworks software was able to show the presence of a reduced metabolite of *N-*methyl bupropion, the *N-*methyl amino alcohol metabolite. The presence of an amino alcohol of *N-*methyl bupropion showed that *N-*methylbupropion (**4**) and bupropion (**3**) are metabolized similarly, by reduction. This amino alcohol was later confirmed as the threo-diastereomer by synthesis of a standard and comparison with the sample chromatogram and fragmentation spectrum. This stereoselective metabolism of *N-*methyl bupropion to *N-*methyl threo-amino alcohol is also seen in the metabolism of bupropion to the threo-amino alcohol. The threo isomer usually predominates over the erythro-isomer in human metabolism. The Michaelis-Menten parameters for *N-*methylbupropion (**4**) were determined. The velocity of formation of product (bupropion) *versus* substrate (*N-*methyl-bupropion) concentration was plotted, Figure 3. *N-*methylbupropion was transformed to bupropion using human liver microsomes with a V_max_ value of 900.4 ± 74 pmol/min/mg HLM and a K_m_ value of 96.8 ± 24.4 μM. The intrinsic hepatic clearance (Cl_i, h_) was found to be 9.28 L/min.

The aqueous stability of *N-*methylbupropion was determined in the same manner as the determination of the aqueous stability of bupropion described [26]. This study was carried out to establish if *N-*methyl bupropion had an improved stability profile over bupropion which could be used to its advantage in the formulation of *N-*methylbupropion prodrug delivery systems. In particular, it was already seen that bupropion had a poor stability profile above pH 5 and any improved profile above this pH would have commercial benefits in formulating *N-*methylbupropion.

**Figure 4:**
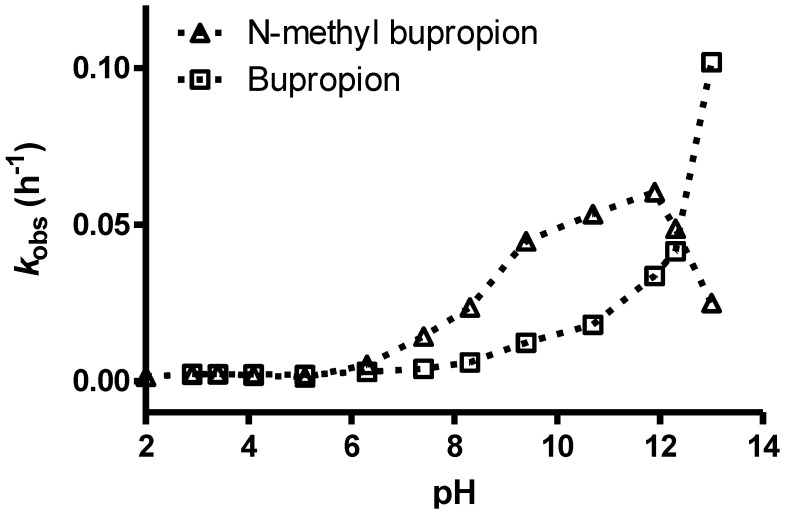
The pH stability profile of *N-*methylbupropion compared to bupropion at 40 °C.

The pH stability profile of bupropion and *N-*methylbupropion at 40 °C is presented in Figure 4. Both bupropion and *N-*methylbupropion follow pseudo-first order decay kinetics in aqueous media. Below pH 5, *N-*methylbupropion and bupropion had similar degradation rates, where they are both protonated and stable. Above pH 5, *N-*methylbupropion degraded faster than bupropion. From pH 6 to 12, the rate of degradation of *N-*methylbupropion increased almost linearly but above pH 12 the rate of degradation decreased quite dramatically. The opposite is true for bupropion where above pH 12, the rate of degradation increases significantly.

The reason for this improved stability of *N-*methylbupropion at high pH is most likely due to the inability of the tertiary amine to form the imine. When bupropion is exposed to high pH it tautomerizes from ketone to enol/enamine to imine, whereas when *N-*methylbupropion is exposed to high pH it may only tautomerize from ketone to enol, the imine cannot form as there is no free proton on the amino group of the tertiary amine. The imine is much more unstable and prone to hydrolysis which is the reason why bupropion’s rate of degradation increases rapidly at high pH. It is unlikely though that *N-*methylbupropion would be exposed to such a pH under physiological or formulation conditions so its improved stability at this pH confers no practical advantage.

**Figure 5:**
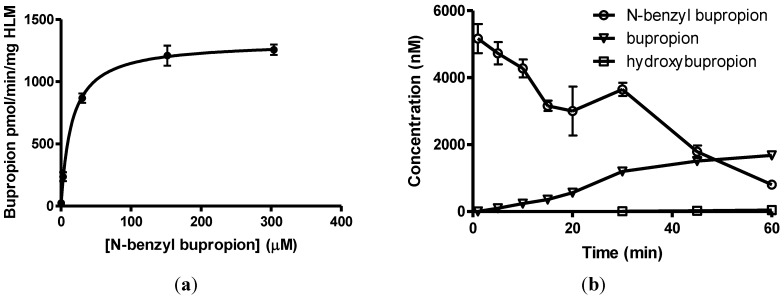
(**a**) Substrate saturation study in pooled human liver microsomes of *N-*benzyl bupropion prodrug (**5**). V_max_ = 1323 ± 42.2 pmol/min/mg HLM, K_m_ = 15.2 ± 2.8 μM, R^2^ = 0.9601, *n =* 6; (**b**) Metabolism of *N-*benzylbupropion (**5**) during a 60 min incubation in HLM.

*N-*benzylbupropion (**5**) was screened using SGF, SIF, HIM, HLM and PHP. Bupropion and new metabolites related to the *N-*benzyl compound were detected in both human intestinal and human liver microsomes. The major product was bupropion while reduced and hydroxylated metabolites were also detected. Reduction of the ketone to the alcohol was most likely the reduced metabolite as this is also the case with bupropion and *N-*methylbupropion. Hydroxylation took place on one of the t-butyl arms and rearrangement occurred. This rearrangement leads to more stable phenylmorphinol ring and was evidenced by the accurate mass spectrum of the metabolite. The ratio of bupropion to hydroxybupropion formation at 60 min was approximately 40:1. The velocity of bupropion formation was quantified by LC-MS and the data was fitted to the Michaelis-Menten equation using least squares no *N-*linear regression analysis, Figure 5a,b. The V_max_ was calculated at 1323 ± 42.2 pmol/min/mg HLM, the K_m_ was 15.2 ± 2.8 µM and the intrinsic hepatic clearance (Cl_i, h_) was found to be 87 L/mg.

Our data suggested that *O*-alkylation of the carbonyl group as well as *N-*alkylation should serve as a useful prodrug for bupropion especially as the resulting *O*-alkyl enol derivative was likely to confer much greater stability over just the *N-*alkylated forms. Dimethyl bupropion (**6**) was synthesized by *O-*methylation of the enolate of *N-*methyl bupropion. This compound was synthesized as a potential multistep prodrug of bupropion. While a number of new metabolites of dimethylbupropion (**6**) were detected, the major metabolite of dimethyl bupropion was *N-*methylbupropion (**4**) formed by *O-*demethylation. Two other minor metabolites were also detected, specifically the hydroxylated metabolite and a metabolite of two mass units greater than the substrate, most likely formed by *O-*demethylation and subsequent formation of the *N-*oxide. The hydroxylated metabolite was also shown to be hydroxylated on one of the *t*-butyl methyl groups. This was evident from the fragmentation pattern of the metabolite. This result indicated that dimethyl bupropion was a good multistep prodrug of bupropion and a potential prodrug for *N-*methyl bupropion. A number of new drug candidates emerged too relating to dimethyl bupropion, the *O-*demethylated, hydroxylated metabolite was a likely prodrug of hydroxybupropion.

During the course of attempting synthesis of an *O-*methyl bupropion, the *N-*benzyl protected *O-*methylated product (**7**) was made as an intermediate. After unsuccessful attempts to remove the benzyl protecting group, it was decided to use the intermediate as is. This compound could potentially be a double prodrug of bupropion if *O-*demethylation and *N-*debenzylation occurred. It was already shown that *N-*debenzylation was a realistic possibility with compound (**5**) and *O-*demethylation is also a common transformation. Prodrug (**7**) was screened in the five metabolic media and was shown to be *O-*demethylated to *N-*benzylbupropion (**5**) in human liver and human intestinal microsomes. New metabolites related to *N-*benzylbupropion (**5**) were also detected.

*N*,*O*-diacetylated bupropion (**8**) contained two potential prodrug groups that are liable to hydrolysis. Therefore this compound was a potential double prodrug of bupropion. Deacetylation at the oxygen would give back the keto-amide, while deacetylation at the amide would give back the amino-enol ester. Amide and ester prodrugs are widely used and have been investigated extensively in the past [39]. Prodrugs of enol esters are not so widely used but this has been used successfully to deliver ketone drugs [31,40,41,42]. Incubation of (**8**) in the media did not result in any activation back to bupropion or its metabolites. It is possible though, that a mono-deacetylation occurred on the oxygen and the keto-amide was returned but the poor ionization of the amide gave poor sensitivity on the detector and this was not detected. Since a deacetylation on the amine would have been much easier detected and was not seen, the tertiary-t-butylated amide is probably too sterically hindered for hydrolysis of the amide by hydrolases.

Screening results of the hydroxyimine of bupropion (**9**) showed that neither bupropion nor any of its metabolites were detected during the course of the metabolism experiment. This was unexpected as oximes are generally susceptible to hydrolysis and are also prone to metabolism by CYP enzymes [30].

**Figure 6:**
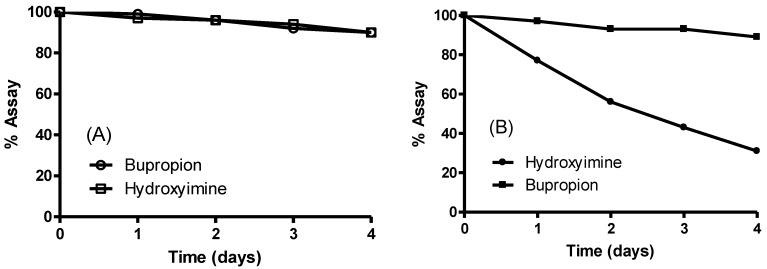
Stability of the hydroxyimine of bupropion (**9**) *vs.* bupropion in (**A**) simulated gastric fluid pH 1.2 at 37 °C and (**B**) phosphate buffered saline solution pH 7.4 at 37 °C.

A short stability study was conducted on the hydroxyimine of bupropion *versus* bupropion in two media at 37 °C to see if the hydroxyimine had greater overall stability. Simulated gastric fluid was chosen to establish if any hydrolysis would occur to the hydroxyimine at low pH but none was detected; bupropion and the hydroxyimine of bupropion had similar stability profiles in this media. At physiological pH though, the hydroxyimine of bupropion had much greater stability than bupropion. This was likely due to intramolecular hydrogen bonding between the proton of the hydroxyl group of the hydroxyimine and the *t*-butyl amine. This added stability of the hydroxyimine may have been the contributing reason why no metabolism to bupropion was occurring. The stability study results are presented in Figure 6.

Although the hydroxyimine has little potential as a prodrug of bupropion, its excellent hydrolytic stability profile and inertness to metabolic enzymes make it a potential drug candidate. Chromatographically, it has the similar retention time as bupropion so has similar lipophilicity.

The oxadiazine (**10**) was designed to be released by possible *N-*dealkylation and subsequent conversion of the resulting oximine intermediate to bupropion. Encouraging results with the *N-*methylated analogue of bupropion suggested that this *N-*dealkyation step was likely proceed efficiently while with the oxime group *O-*alkylated it was hope that hydrolysis of this group would occur more efficiently than with oxime (**9**). However, screening of the oxadiazine (**10**) in each of the five metabolic media resulted in the formation of the oxime (**9**) with little evidence as before of its conversion to bupropion. The oxadiazinone (**11**) was resistant to all of the screening conditions utilised.

Oxazolone (**12**) should be prone to acid or base hydrolysis as the carbonyl group in this molecule should be electrophilic. The possibility of ring opening and further dealkylation could return bupropion. The concentration of oxazolone was decreasing over the course of the incubations, but there was no bupropion or metabolites thereof detected.

Although compound (**14**) was the unexpected product from our attempt to synthesize the enolalkylated derivative of this compound, it was nevertheless envisaged that bupropion could be regenerated following *O*-dealkylation, hydrolysis of the carbamate group, elimination of HCl to form the enol and subsequent enol to keto tautomerism was likely to furnish bupropion. However the screening of (**14**) in each media did not generate bupropion or any of its expected metabolites.

The most likely prodrug candidate from all compounds tested above was *N-*methylbupropion. In order to determine if *N-*methyl bupropion was truly a prodrug of bupropion its basic CNS pharmacological profile was characterised.

Since bupropion’s activity in depression was known to be related to its dual reuptake inhibition of dopamine and norepinephrine, these two transporter systems were evaluated. Serotonin inhibition was also evaluated in case *N-*methyl bupropion showed some triple reuptake inhibition including the serotonin transport system. Bupropion is also known to act upon nicotinic acetylcholine receptors, which lead to its activity as a smoking cessation agent. Therefore activity at nicotinic acetylcholine receptors was also evaluated. Finally, activity was evaluated was at the muscarinic acetylcholine receptor. Activity at this receptor may indicate use as an anti-Parkinsons therapeutic [43].

A summary of the pharmacological screen is presented in Table 2. IC_50_ values were calculated using non*-*linear least squares regression, inhibition constants (*K_i_*) were calculated using the equation of Cheng and Prusoff and the Hill coefficient (n_H_) defines the slope of the competitive binding curve. Hill coefficients significantly different from 1.0 may suggest that the binding displacement does not follow the law of mass action with a single binding site.

**Table 2:** Pharmacology of *N-*methyl bupropion compared to bupropion.

*N-*methylbupropion			Target	Bupropion		
IC_50_ (μM)	*K_i_* (μM)	n_H_		IC_50_ (μM)	*K_i_* (μM)	n_H_
39.3	12.3	0.957	Muscarinic non *-*selective, central	86.5	27	1.42
71.6	49.2	0.836	Nicotinic acetylcholine	>100	-	-
3.71	2.95	0.924	Dopamine active transporter (DAT)	<1	-	-
57.9	54.4	0.764	Norepinephrine transporter (NET)	19.9	19.8	0.835
>100	-	-	Serotonin transporter (SERT)	>100	-	-

The pharmacological screens of *N-*methylbupropion *versus* bupropion showed that *N-*methyl-bupropion is less active than bupropion as a dopamine and norepinephrine reuptake inhibitor and could be used as a potential prodrug for these therapeutic targets. *N-*methylbupropion is more active than bupropion at the nicotinic acetylcholine receptors and has potential as a new therapeutic in the smoking cessation area, although further characterization into which specific nicotinic receptors should be established. Both *N-*methylbupropion and bupropion have little effect on the serotonin transporter system.

## 4. Conclusions

A successful tool was developed for *in vitro* screening of potential prodrugs of bupropion which utilized five different media that a drug substance is likely to encounter via the oral route of delivery. This screening tool has the potential to be used generically to evaluate any drug or prodrug stability for oral delivery.

Two interesting prodrugs of bupropion arose from this work namely the, *N-*methylbupropion (**4**) and *N-*benzylbupropion (**5**). An oxadiazine (**10**) bioprecursor of the hydroxyimine (**9**) of bupropion was found. The hydroxyimine (**9**), while not a prodrug in itself, showed excellent stability and may be a candidate for further screening in biological assays. The *N-*methyl, *N-*benzyl and oxadiazine were *N-*dealkylated by human liver and human intestinal microsomes indicating that the tertiary amine is susceptible to oxidation by the CYP or FMO enzymes. It is therefore quite likely that other *N-*alkyl groups such as ethyl, propyl or codrugs linked via an alkyl bridge could be appended to bupropion and would be successfully *N-*dealkylated through metabolism.

Two potential multistep prodrugs of bupropion were realized, *N*,*O*-dimethylbupropion (**6**) and *N-*benzyl-*O-*methylbupropion (**7**), which in themselves are bioprecursors of *N-*methyl- and *N-*benzyl- bupropion, respectively. Therefore enol-ethers are susceptible to the oxidative enzymes of the CYP and FMO family. A number of new metabolites of these prodrugs were identified with potential pharmacological activity.

*N-*benzylbupropion (**5**) was evaluated for its enzyme kinetics using human liver microsomes. It was transformed to bupropion at a higher rate than *N-*methylbupropion and it had a higher enzyme-substrate affinity. A number of new metabolites relating to *N-*benzylbupropion were found, namely the reduced form *N-*benzylamino alcohol and a hydroxylated *N-*benzylbupropion. These metabolites could also have potential pharmacotherapies. *N-*benzylbupropion may also be a good prodrug candidate of bupropion.

*N-*methylbupropion (**4**) was evaluated for its enzyme kinetics, pharmacology and stability. *N-*methyl bupropion was also shown to be reduced to the amino alcohol, this new metabolite could also have potential pharmacotherapies. *N-*methylbupropion was shown to have a similar stability profile to bupropion below pH 5. The pharmacological profile of *N-*methylbupropion was screened in a DAT, NET, SERT, nAChR and nMAChR assay. It was shown to be less active than bupropion in DAT and NET assays. *N-*methylbupropion was therefore deemed a good candidate as a potential prodrug of bupropion for an *in vivo* proof of concept study.

## Author Contributions

P.M.O.B. performed the chemical syntheses, characterization, analysis and wrote the manuscript. J.J.W. and J.F.G. coordinated the studies, supervised the experiments, reviewed the data generated and reviewed the manuscript. R.W. acted as an external consultant to the project and contributed to the coordination of experiments. All authors approved the final manuscript.

## Supplementary Files

Supplementary File 1 Supplementary Information (PDF, 220 KB)
